# Phosphatidylserine receptors TIM-1 and AXL mediate tick-borne Powassan virus entry

**DOI:** 10.1016/j.isci.2025.113930

**Published:** 2025-11-03

**Authors:** Maria Daskou, Anne K. Zaiss, Arjit Vijey Jeyachandran, Kari-Ann Takano, Ryan L. Kan, Ramya Paravastu, Ephrem Gerald, Nivedha Satheeshkumar, Jennifer Rios-Rodriguez, Brandy Russell, Aaron C. Brault, Aparna Bhaduri, Gustavo Garcia, Kouki Morizono, Vaithilingaraja Arumugaswami

**Affiliations:** 1 Department of Molecular and Medical Pharmacology, University of California, Los Angeles, Los Angeles, CA, USA; 2 Department of Medicine, University of California, Los Angeles, Los Angeles, CA, USA; 3 Department of Biological Chemistry, University of California, Los Angeles, Los Angeles, CA, USA; 4 Centers for Disease Control and Prevention (CDC), Division of Vector-Borne Diseases, Fort Collins, CO, USA; 5 Eli & Edythe Broad Center of Regenerative Medicine and Stem Cell Research, UCLA, Los Angeles, CA, USA; 6 California Nanosystems Institute, UCLA, Los Angeles, CA, USA

**Keywords:** Pharmacology, Molecular biology, Virology

## Abstract

Powassan virus (POWV) is an emerging tick-borne neurotropic human pathogen. Currently, there are no approved medications or vaccines available. The goal of this study is to investigate the receptor usage for POWV, which can be a potential therapeutic target. For this purpose, we used cell culture-based models including neuronal cells, 293T cells stably expressing different viral entry receptors, and induced pluripotent stem cell (iPSC)-derived cortical organoids, as well as *in vivo* studies in wild-type C57BL/6 mice. Among the receptors studied, phosphatidylserine (PtdSer)-recognizing TIM-1 and AXL receptors facilitated higher infection. To further validate our findings, a neutralization assay was performed in which the soluble form of the TIM-1 receptor efficiently blocked infection. In addition, we demonstrated that PtdSer receptor-expressing cells in cortical organoids and mouse brain tissues were infected with the virus. We conclude that PtsSer moieties on POWVs’ surface facilitate viral entry through the cellular TIM-1 and AXL receptors.

## Highlights

• TIM-1 and AXL surface receptors facilitate POWV and DTV cell entry *in vitro*
• Blockage of TIM-1 and AXL resulted in robust neutralization of POWV and DTV virus infection
• Potential role of PtdSer receptors in POWV and DTV entry *in vivo*

## AB_883882Graphical abstract

**Figure:**
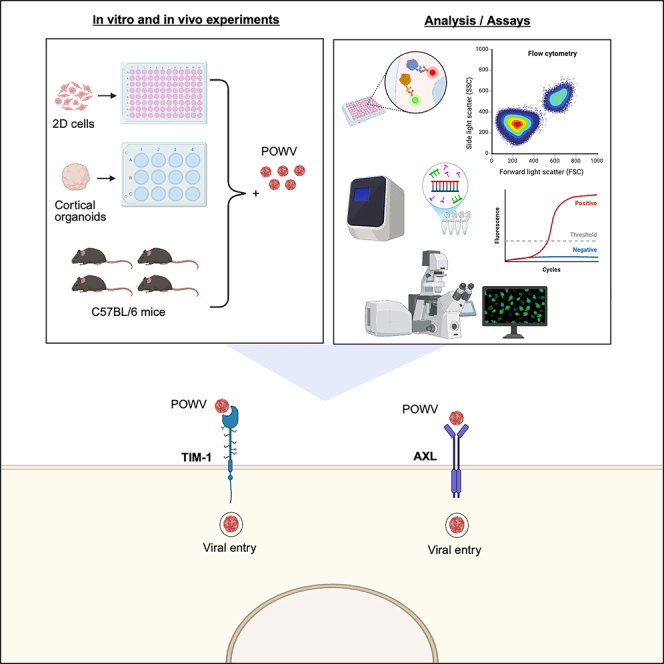

## Introduction

Due to ongoing climate change, the rising tick population will only continue to expand in the upcoming years due to geographic expansion.^1^ As a result, the viral, bacterial, and other pathogens that these vectors harbor are at increasing risk of transmission to the human population. An emerging tick-borne pathogen, Powassan virus (POWV), is endemic in the Northeast and Midwest United States and into Canada and causes severe neurological illnesses.^2^^,^^3^ POWV belongs to the Flaviviridae family, which has a single-stranded RNA genome of about 10.8 kb; this encodes a single large open reading frame composed of seven nonstructural (NS) and three structural proteins.^3^ The structural protein capsid assembles to form a virion particle with icosahedral morphology and cubic symmetry.^4^

There are two recognized viral lineages: the prototype lineage I POWV and the lineage II deer tick virus (DTV).^5^ DTV isolates are further separated into clades depending on the geographic area of isolation.^6^ POWV and DTV are maintained within different species of ixodid tick vectors (hard ticks) and small mammalian hosts, such as skunks, weasels, woodchucks, squirrels, and others.^7^ POWV has been isolated from various tick species, including *Ixodes cookei*, *Ixodes marxi*, and *Ixodes spinipalpus*, whereas DTV has primarily been detected in *Ixodes scapularis* ticks.^8^^,^^9^ Both viral lineages can cause human disease,^10^ but recent evidence suggests the emergence of the DTV subtype.^9^^,^^11^^,^^12^ Most of the newly detected POWV infections have been identified as DTV^13^^,^^14^^,^^15^ or were associated with exposure to *I. scapularis* tick species, the vector for DTV.^11^ The growing population and expanding geographic range of *I. scapularis* ticks,^11^ combined with their higher tendency to bite humans compared to *I. cookei*, the primary vector for POWV lineage I,^3^ suggest a link between the spread of *I. scapularis* and the rising incidence of DTV cases.^6^^,^^16^ However, several caveats must be considered when interpreting this trend. These include potential sampling bias, differences in tick host preferences and enzootic transmission cycles between lineages, ecological or geographic barriers to spread, and the possibility of underreported human cases. Additionally, since the two lineages are serologically indistinguishable, current diagnostics may fail to differentiate them, obscuring true lineage-specific prevalence. It is also important to identify the primary *Ixodes* tick species involved in maintaining each lineage in nature, as this may further influence observed patterns.

Antibodies against POWV have been detected in the serum of deer, groundhogs, squirrels, mice, or other rodents.^7^^,^^17^ Ticks are exposed to POWV following different transmission mechanisms, including horizontal (non-hereditary), vertical (parent-progeny), and transstadial (passage through life stages) transmission by *Ixodes* ticks.^18^^,^^19^ Horizontal transmission can occur through blood meals from infected animals or cofeeding (transmission between infected and uninfected vectors feeding simultaneously on the same host).^20^ In addition, it has been shown that POWV could be transferred to a tick through the transovarial route.^21^^,^^22^ Thus, the bite of an infected tick can transmit the virus to a human host. Within as little as 15 min^23^ to 3 h of a tick bite, initial replication of POWV in humans takes place proximal to the site of viral inoculation.^24^ Macrophages and fibroblasts near the site of a tick bite are early cellular targets of POWV.^24^ Immune cells, such as macrophages and dendritic cells, are believed to transport the virus to the local draining lymph nodes, where the virus continues to amplify. Replication in lymph nodes is the major indicator of an early viremic stage of infection, where patients experience a nonspecific flu-like illness.^25^ This is followed by viral invasion of the central nervous system (CNS) through the blood-brain barrier (BBB). The exact neuroinvasive mechanism is unknown, although studies have found that tumor necrosis factor alpha and the brain capillary endothelium may be implicated.^26^ In the CNS, POWV primarily targets neurons, causing neuronal cell death and changes in their dendrite structures.^27^^,^^28^ The POWV effect on neurons can be a combination of direct killing and immune-mediated adverse effects.^29^ The virus has also been shown to induce weak antiviral inflammatory responses and failed to induce apoptosis in human neural stem cell-derived neuron-astrocyte co-cultures,^29^ suggesting it may have the ability to evade innate immune response in these cells. Furthermore, POWV infects endothelial cells and pericytes,^28^ impacting the integrity of the BBB. CNS invasion by the virus results in clinical diseases such as encephalitis, specifically rhombencephalitis, and meningitis, which can lead to seizures.^30^ This viral encephalitis is lethal in 11% of cases, and recovering patients often experience long-term neurological sequelae.^3^

Flaviviruses have been shown to utilize several host receptors in entry, including α_v_β_3_ integrins, C-type lectin receptors (CLR), phosphatidylserine (PtdSer) receptors TYRO3 and TIM (T cell immunoglobulin and mucin domain), AXL receptor tyrosine kinase (AXL), and MER receptor tyrosine kinase (MERTK).^31^ The tyrosine kinase receptors TYRO3, AXL, and MERTK are collectively known as TAM receptors. Entry into dendritic cells, early targets of flaviviruses, is primarily mediated by the C-type lectin receptor, dendritic cell-specific intercellular adhesion molecule-3-grabbing non-integrin (DC-SIGN), while CNS cell entry is often mediated by CLRs and TIMs.^31^^,^^32^ TIM receptors specifically have been widely shown to regulate innate and adaptive immunity, especially T cell responses; in the viral entry context, TIM directly interacts with viral envelope PtdSer, allowing for internalization.^32^ Despite existing knowledge of flaviviral entry receptors, the receptors that POWV engages with to enter host cells have not been described yet. POWV envelope (E) protein functions in host receptor recognition and receptor-mediated fusion entry.^33^

In this study, we investigated cell surface receptors critical for POWV and DTV entry into their host cells. By using a combination of cell culture-based models, including 293T cells stably expressing different viral entry receptors, induced pluripotent stem cell (iPSC)-derived cortical brain organoids, and *in vivo* studies in immunocompetent mice, we were able to reveal PtdSer receptor family TIM-1 and AXL as entry receptors for POWV and DTV viruses.

## Results

### POWV and DTV viruses efficiently establish neuronal cell infection

**Figure 1:**
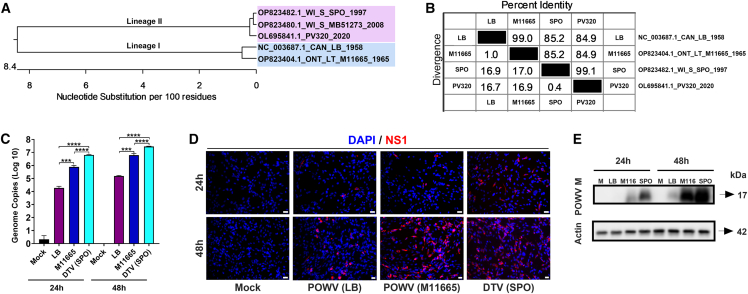
Genomic sequence analysis and infectious U118 neuronal cell culture system (A) Phylogenetic analysis of five Powassan viral genome sequences from known lineage I and II strains. GenBank accession numbers are provided along with strain information including location and year of isolation. Phylogenetic tree was constructed using the neighbor-joining method with pairwise distance estimation following ClustalW alignment program. (B) Matrix panel shows the percent identity in the upper right and nucleotide divergence in the lower left of aligned Powassan viral genomic sequences (ClustalW program, DNASTAR Lasergene MegAlign 15). (C) Graph shows the RT-qPCR analysis of POWV (LB), POWV (M11665), and DTV (SPO) genome replication in U118 cells 24 and 48 hpi. The *y* axis shows genome copies per 50 ng of total RNA in log scale. Error bars represent the SD of three biological replicates. Student’s *t* test. ∗*p* < 0.01, ∗∗*p* < 0.001, ∗∗∗*p* < 0.0001, ∗∗∗∗*p* < 0.00001. (D) Representative immunofluorescence images of mock and LB-, DTV- (M11665), and DTV- (SPO) infected U118 neuronal cells detected by viral NS1 at 24 and 48 hpi (scale bars, 25 μm). Red, NS1 viral protein; blue, DAPI. (E) Western blot analysis of infected cell lysates probed with the anti-M protein antibody targeting LB, DTV (M11665), and DTV (SPO) at 24 and 48 hpi. Cells were infected with each virus at an MOI of 1. Two independent experiments were conducted. Representative data are presented.

POWV and DTV are tick-borne neurotropic viruses belonging to lineage I and lineage II, respectively.^26^^,^^34^ In this study, we utilized lineage I strains LB and M11665 as well as lineage II strain SPO for infection studies. Phylogenetic analysis, along with percent identity and divergence, is shown in Figures 1A and 1B. Strains within each lineage are closely related, sharing 99% nucleotide sequence identity, whereas strains from different lineages share 85% identity (Figure 1B). To compare replication kinetics between lineages, we conducted infection studies using a neuronal infectious cell culture system at an MOI of 1. Utilizing RT-qPCR analysis of the infected cells at 24 and 48 hours post-infection (hpi), we generated replication kinetics for each virus, confirming an active infection of the brain cells (Figure 1C). Viral load in the infected cells significantly increased with time for all three viruses. Interestingly, the POWV (LB) strain exhibited a 2-log lower replication level, which was statistically significant, compared to the DTV (SPO) strain at these two time points. (Figure 1C). It is important to note that these historical isolates were recovered from clinical or tick samples following at least one passage in suckling mouse brain. However, the lineage I LB and M11665 strains underwent four and two passages in suckling mouse brain, respectively, before amplification in Vero cells, compared to just one passage for the SPO isolate.^35^ Moreover, we observed that the lineage II DTV (SPO) strain replicated significantly more than both lineage I strains. Additionally, infection of these neuronal cells was confirmed by fluorescent microscopy through detection of the NS1 viral protein at 24 and 48 hpi (Figure 1D). To further validate our results, we performed a western blot analysis by probing for viral structural protein matrix (M). The results showed expression of the M protein by POWV (M11665) and DTV (SPO) isolates at both 24 and 48 hpi, while for the POWV (LB) strain, the M protein was only detected at 48 hpi due to lower replication level (Figure 1E). Taken together, these results show that we have established an infectious cell culture system and assays to evaluate the replication of both lineage I and II viruses.

### Mapping cell entry receptors for POWV and DTV

The cellular receptors used by POWVs to enter the host cells are currently unknown. It has been shown that many flaviviruses can use DC-SIGN, AXL, and TIM-1 (otherwise referred to as hepatitis A virus cellular receptor 1 [HAVCR1]) as entry receptors to infect their target cells.^31^ For this reason, we used 293T cells stably expressing DC-SIGN, AXL, or TIM-1 receptors in comparison with 293T wild-type (293T WT) cells that do not naturally express these receptors.^36^ DC-SIGN receptor is a member of the C-type lectin receptors and is highly expressed on the surface of macrophages and dendritic cells.^37^ AXL is a member of the TAM family of receptor tyrosine kinases and is expressed in multiple cell types including astrocytes and dendritic cells; it plays important roles in cell growth and anti-inflammatory responses.^38^^,^^39^ TIM-1 is a PtdSer receptor that is expressed in Th2 cells, mast cells, regulatory B cells, and brain cells such as neurons, astrocytes, and microglia.^40^^,^^41^ Both AXL and TIM-1 recognize PtdSer moieties on the surface of cell plasma membranes.^36^^,^^42^

**Figure 2:**
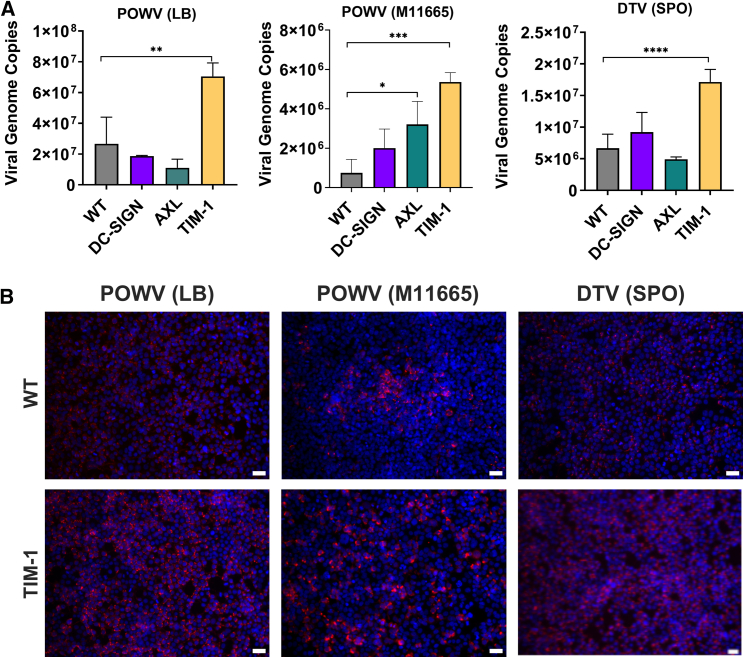
Infection of 293T cells stably expressing AXL, DC-SIGN, and TIM-1 receptors (A) Graph shows RT-qPCR analysis of indicated 293T cells infected with each virus (MOI of 1) at 24 hpi. The *y* axis shows genome copy numbers (log 10) detected in 50 ng of input RNA. Error bars represent the SD of three biological replicates. Student’s *t* test. ∗*p* < 0.01, ∗∗*p* < 0.001, ∗∗∗*p* < 0.0001, ∗∗∗∗*p* < 0.00001. (B) Immunofluorescence images of 293T WT and 293T-TIM-1 cells infected with each virus at 24 hpi (scale bars, 25 μm). Red, NS1 viral protein; blue, DAPI. Representative data from two independent experiments were presented.

To evaluate the usage of host cell receptors by these viruses, we utilized human 293T WT cells as well as 293T cells stably expressing DC-SIGN, AXL, or TIM-1 receptors. The establishment of these stable cell lines and the characterization of exogenous receptor protein expression in each have been previously described.^36^ Each cell line was inoculated with one of the three viruses at an MOI of 1, and at 24 hpi, the cells were harvested for RT-qPCR and immunofluorescence analysis (Figure 2). RT-qPCR showed that the POWV (LB) strain had a significant 2.6-fold viral genome copy number increase in TIM-1-expressing cells compared to WT cells (*p* < 0.001). Interestingly, POWV (M11665) showed significantly higher levels of infection in AXL- and TIM-1-expressing cells—approximately 4-fold (*p* < 0.01) and 7-fold (*p* < 0.0001), respectively—compared to WT cells. Additionally, this strain replicated more than 2-fold higher in 293T-DC-SIGN cells relative to WT cells. The infection pattern for the DTV (SPO) virus was similar to that of POWV (LB), with only 293T-TIM-1 cells supporting a significantly higher level of replication compared to the WT cells (3-fold, *p* < 0.00001) (Figure 2A). A caveat is that all these viruses replicated to levels greater than 10^6^ in 293T WT cells, suggesting that additional host receptors are involved in POWV entry. Our results indicated that, among the receptors tested, TIM-1 expression facilitated efficient viral entry and replication (Figure 2A). Furthermore, fluorescent microscopy analysis confirmed that 293T-TIM-1 cells had higher levels of viral infections (Figure 2B).

### Expression of PtdSer receptors in Vero E6 and U118 cells and neutralization assay using a soluble form of TIM-1 PtdSer receptor, sTIM-1

**Figure 3:**
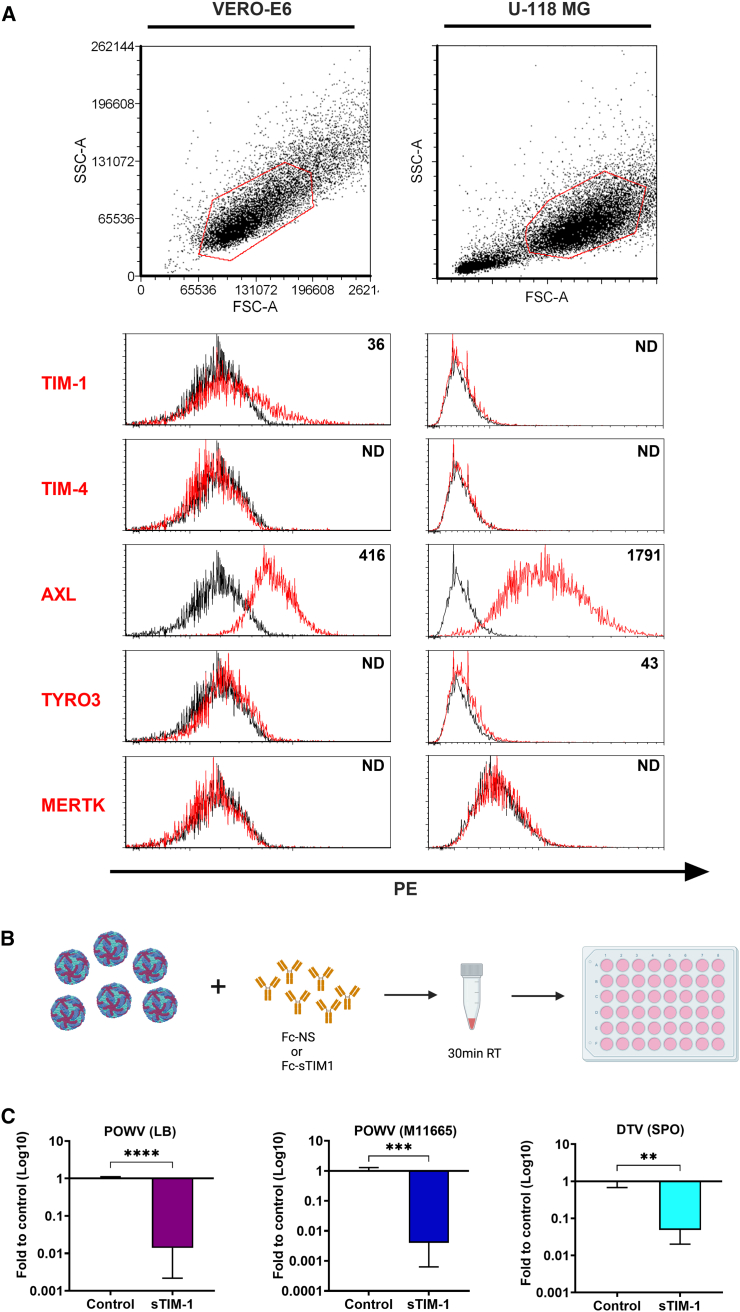
Expression analysis of TIM-1, TIM-4, AXL, TYRO3, and MERTK on Vero E6 and U118 cells and viral entry-neutralization assay (A) FSC/SSC plot with gating and histograms from flow analysis are shown. Black line shows cell staining by control goat antibody, while the red line represents staining by antibodies against specific PtdSer receptors. The value indicated in the histogram corresponds to the expression level of each receptor protein calculated by subtracting the mean fluorescent intensity of the control antibody-stained cells from that of the cells stained with an antibody specific to a PtdSer receptor. ND, not detected. Note: TIM-1 and AXL expression on the Vero E6 cell surface was found. TIM-4, TYRO3, and MERTK were not expressed. On the U118 cell surface, only AXL expression was found. (B) Schematic representation of neutralization assay using 100 plaque-forming units of virus incubated with soluble forms of TIM-1 receptor Fc-sTIM1 (sTIM1dMLDR801) or control protein Fc-NS (NCFcCQ R801). (C) Graphs show fold inhibition of indicated viral replication following neutralization with sTIM1dMLDR801. Error bars represent the SD using three biological replicates. Student’s *t* test. ∗*p* < 0.01, ∗∗*p* < 0.001, ∗∗∗*p* < 0.0001. Representative data from two independent experiments were provided.

Since TIM-1 significantly increased the infection for all three viruses (Figure 2A), we performed a follow-up neutralization study. TIM-1 can bind directly to the PtdSer of the viral envelope protein and facilitate viral entry.^36^ We performed a viral entry-neutralization assay using a soluble form of TIM-1 receptor Fc-sTIM1 (sTIM1dMLDR801) or control protein Fc-NS (NCFcCQ R801), in Vero E6 cells. The sTIM1dMLDR801 reagent is capable of competitively blocking PtdSer-dependent infection of enveloped viruses mediated by PtdSer-recognizing receptors such as TIM-1, TIM-4, and AXL.^43^ Initially, we evaluated the expression of PtdSer receptors such as TIM-1, TIM-4, AXL, MERTK, and TYRO3 in naive Vero E6 and U118 cells by flow cytometry analysis. The results showed that both TIM-1 and AXL are expressed in Vero E6 cells, whereas only AXL is expressed in U118 cells. Other PtdSer receptors capable of mediating viral infection were not detected in either cell type (Figure 3A). For the viral entry-neutralization assay, 100 plaque-forming units of each virus were incubated for 30 min with 10 μg/mL of sTIM1dMLDR801 or control NCFcCQ R801 at room temperature, followed by inoculation onto Vero E6 cells (Figure 3B). The infected cells were cultured for the next 24 h at 37°C in a tissue culture incubator and subsequently lysed for harvesting total RNA. The results from RT-qPCR analysis at 24 hpi showed that in Vero E6 cells, the soluble TIM-1 receptor neutralized infection for all tested viruses, demonstrating that TIM-1 and AXL are important entry receptors for PtdSer-dependent infection of POWV and DTV (Figure 3B).

### PtdSer receptor expression pattern in DTV-infected human cortical organoids and mouse brain

**Figure 4:**
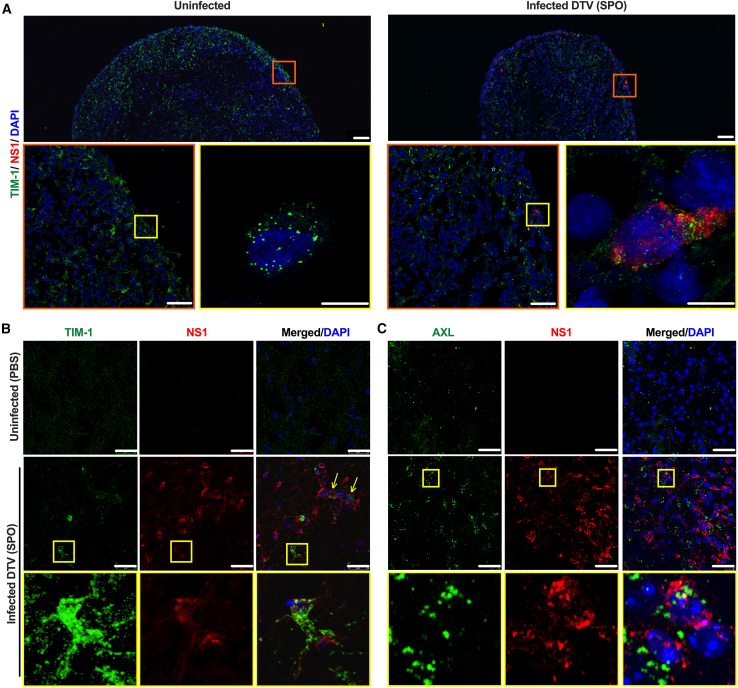
PtdSer receptor expression pattern in DTV-infected human cortical organoids and mouse brain tissues (A) Confocal images of uninfected and DTV (SPO)-infected cortical organoids show that TIM-1-expressing cells are positive for viral infection as detected by NS1. Insets show higher magnification of an infected TIM-1-positive cell (scale bars: 5 μm in [top] and 10 μm and 75 μm in [insets]). (B) Confocal images of uninfected (PBS) and DTV-infected (6 dpi) C57/BL6 mouse brain tissues depict TIM-1 and viral NS1 expression patterns. Note: Uninfected brain cells from PBS control show uniform granular distribution of TIM-1. Infected brain cells show patches of prominent TIM-1 aggregates (scale bars, 25 μm). Arrows denote DTV-infected cells in a blood vessel expressing TIM-1. Insets show a higher magnification of DTV-infected TIM-1-positive glial cell. Green, TIM-1 surface protein; red, NS1 viral protein; blue, DAPI. (C) Utilizing confocal microscopy, images of uninfected (PBS) and DTV-infected (6 dpi) mouse brain tissues depicting AXL and viral NS1 expression patterns were acquired (scale bar, 25 μm). Insets show a higher magnification of DTV-infected AXL-positive cell. Green, AXL surface protein; red, NS1 viral protein; blue, DAPI.

After demonstrating PtdSer receptor utilization by POWV and DTV in 2D cell culture models, we proceeded with verification of TIM-1 expression in human cortical organoids and mouse brain tissues—biologically relevant model systems to simulate POWV brain infection conditions. DTV (SPO) was selected for these experiments because most recently detected POWVs, including the 2020 human clinical isolate PV320 (Figure 1), belong to the DTV lineage.^6^^,^^16^ Moreover, our results in the cell culture models showed that DTV (SPO) established robust infection. Therefore, DTV-infected human cortical organoids and collected mouse brain tissues were used to study the expression of TIM-1 and NS1 viral protein with immunofluorescent staining and immunohistochemistry, respectively (Figure 4). Confocal imaging of uninfected (mock) and DTV (SPO)-infected cortical organoids confirmed that TIM-1 surface receptor-positive cells are infected by DTV based on NS1 protein detection (Figure 4A). Similarly, in brain tissues from control and DTV (SPO)-infected mice stained for host TIM-1 and viral NS1 protein, confocal microscopy revealed DTV infection of TIM-1-positive cells (Figure 4B). Interestingly, uninfected mouse brain cells had uniform granular distribution of TIM-1, whereas infected brain tissues had a prominent coarse aggregated TIM-1 distribution pattern (Figure 4B), which was not observed in the infected human brain organoids, likely reflecting receptor redistribution mediated by *in vivo* immune responses and inflammation. In addition, brain tissues from control and DTV (SPO)-infected mice were immunostained for host AXL and viral NS1 protein. The results from confocal microscopy showed DTV infection in AXL-positive cells (Figure 4C). Taken together, these results demonstrate that TIM-1 and AXL can function as an entry receptor for POWVs in susceptible brain target cells.

## Discussion

POWV and DTV are known to infect neurons, astrocytes, endothelial cells, and pericytes in the brain. However, the receptors used by these viruses to enter these cells are unknown.^28^^,^^29^ As a starting point, we chose to study DC-SIGN, AXL, and TIM-1 as potential POWV and DTV entry receptors as these are common flaviviral entry receptors used by Zika, dengue, and West Nile viruses. Enveloped viruses can infect target cells through carbohydrate-dependent interactions between their envelope proteins and DC-SIGN,^44^ whereas AXL- and TIM-1-mediated entry relies on recognition of PtdSer on the viral envelope. Previous studies have revealed that during enveloped viruses’ infection, the Gas6 serum protein acts as a bridge between the envelope PtdSer and the AXL surface receptor.^36^^,^^45^^,^^46^ In contrast to AXL, TIM-1 can bind PtdSer directly,^46^ since the IgV domain of TIM-1 contains a high-affinity PtdSer-binding site.^47^ This mechanism of interaction allows usage of TIM-1 and AXL as an entry receptor by a broad range of enveloped viruses. Previous studies have confirmed that TIM-1 serves as a receptor for various viruses like Ebola,^48^ hepatitis A,^49^ chikungunya,^50^ dengue,^51^ and Zika.^43^ Moreover, another recent study showed that TIM-1 is a functional entry factor for the tick-borne encephalitis virus, a related neurotropic flavivirus.^52^

**Figure 5:**
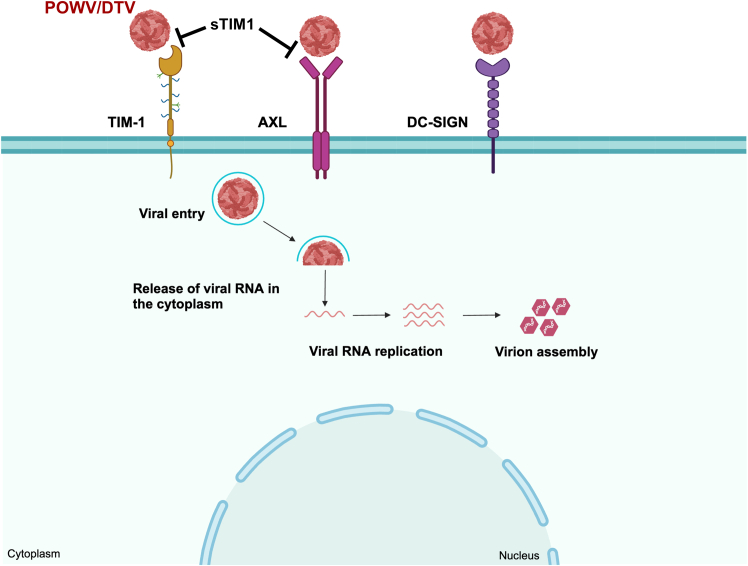
A hypothetical model of TIM-1 and AXL functions in viral entry POWV and DTV viruses can interact directly with TIM-1 or AXL through binding of its IgV domain with the viral envelope PtdSer, leading to fusion with the plasma membrane and entry into the cytoplasm of susceptible target cells. Usage of a soluble form of TIM-1 protein can prevent infection. Additional entry receptor DC-SIGN is depicted.

In the present study, we showed that TIM-1 and AXL are important entry receptors for POWV and DTV infection *in vitro* (Figure 5). Additionally, we confirmed that TIM-1- or AXL -expressing cells in mouse brain tissues are susceptible to DTV infection, indicating a critical role of PtdSer receptors in viral entry *in vivo*. TIM-1 and AXL knockout in animal or organoid model systems can be used to further validate our results. However, the gene knockout of an important cellular protein can yield non-specific and loss-of-function effects on cells, which can affect the interpretation of viral entry results. Interestingly, using a 293T-based overexpression system, we showed that POWV (M11665) could also use DC-SIGN and AXL to infect host cells. Although no specific cell-adaptive mutations have been described for this strain due to passage history, it is possible that cell-adaptive mutations acquired during passaging may have conferred an expanded receptor usage phenotype. However, further studies are needed to compare this strain with contemporary isolates that have rigorously curated genomic sequence data prior to passaging in animals and/or cell cultures.

Our results shed a new light on POWV- and DTV-host interactions. We established the usage of human cortical organoids to model POWV and DTV infection and host responses in the human brain. Overall, the cell culture and animal model systems developed in this study can serve as tools for the identification and verification of additional viral entry receptors that can then be therapeutically targeted against these emerging tick-borne viruses. Based on our results, we suggest that TIM-1 and AXL blockage can be used as a potential therapeutic approach against POWV and DTV viruses alone or in combination with other antiviral strategies.

### Limitations of the study

Our study demonstrates TIM-1 and AXL as entry receptors for POWV, which we assessed using a combination of overexpression and neutralization assays. A limiting factor is that we did not perform neutralization assays *in vivo*. POWV, like other flaviviruses, may be able to utilize additional receptors to enter the cells *in vivo.* Also, it is important to note that POWV LB is a highly passaged historic isolate (including multiple passages in suckling mouse brains) that is unlikely to represent currently circulating lineage 1 strains. Thus, the use of the low-passage Powassan viral strains directly isolated in cell culture without undergoing cell-adaptive mutations can further help understand cell tropism and relevant disease mechanisms.

## Resource availability

### Lead contact

Requests for further information and resources should be directed to and will be fulfilled by the lead contact, Vaithilingaraja Arumugaswami (varumugaswami@mednet.ucla.edu).

### Materials availability

This study did not generate new unique reagents.

### Data and code availability

• All data reported in this manuscript will be shared by the lead contact upon request.
• This paper does not report original code.
• Any additional information required to reanalyze the data reported in this study is available from the lead contact upon request.

## Acknowledgments

We are grateful to Barbara Dillon, UCLA High Containment Program Director, for BSL3 work. We are also grateful to Nikhil Chakravarty for editing the manuscript. Figures 3A and 5 were created with BioRender.com. This study is partly supported by 10.13039/100000002National Institutes of Health awards 1R01EY032149-01, 5R01AI163216-02, 1R01EY036572-01, and 1R01DK132735-01 to V.A.

## Author contributions

M.D., conceptualization, methodology, data analysis, interpretation, original draft preparation, and editing; A.K.Z. and J.R.-R., methodology and editing; A.V.J., K.-A.T., R.L.K., R.P., E.G., and N.S., methodology; G.G.J., project administration, methodology, visualization, and editing; B.R., A.C.B., A.B., and K.M., resources, data analysis, and interpretation; V.A., conceptualization, data analysis, and interpretation, review, editing, and final approval of manuscript.

## Declaration of interests

The authors declare no competing financial interests.

## STAR★Methods

### Key resources table

**Table:** 

REAGENT or RESOURCE	SOURCE	IDENTIFIER
**Antibodies**		

Powassan virus prM protein antibody	GeneTex	Cat#GTX132055; RRID: AB_2886551
Powassan Virus NS1 Monoclonal Antibody (3681)	Thermo Fisher Scientific	Cat#MA5-47516; RRID: AB_2942497
Goat anti-Mouse IgG (H + L) Cross-Adsorbed Secondary Antibody Alexa FluorTM 555	Thermo Fisher Scientific	Cat#A-21422; RRID: AB_141822
TIM-1 Polyclonal Antibody	Thermo Fisher Scientific	Cat#PA5-20244; RRID: AB_11152868
Goat anti-Rabbit IgG (H + L) Cross-Adsorbed Secondary Alexa FluorTM 488	Thermo Fisher Scientific	Cat#A-11008; RRID: AB_143165
Human Axl Antibody	bio-techne	Cat#AF154; RRID: AB_354852
Human Tyro3/Dtk Antibody	bio-techne	Cat#AF857; RRID: AB_883882
Human Mer Antibody	bio-techne	Cat#AF891; RRID: AB_355691
Human TIM-1/KIM-1/HAVCR Antibody	bio-techne	Cat#AF1750; RRID: AB_2116561
Human TIM-4 Antibody	bio-techne	Cat#AF2929; RRID:AB_2240431
Donkey Anti-Goat IgG H&L (PE) preabsorbed	Abcam	Cat#Ab7004; RRID: AB_955479
Normal Goat IgG	bio-techne	Cat#AB-108-C; RRID: AB_354267

**Bacterial and virus strains**		

POWV strain LB (LOT TC00580 WSV)	CDC	N/A
DTV WI_S_SPO_1997 [DTV (SPO)]	CDC	N/A
Powassan Deer Tick virus (ONT_LT_M11665_1965; M11665)	BEI	Cat#NR-51182

**Chemicals, peptides, and recombinant proteins**		

Eagle’s Minimum Essential Medium (MEM)	Corning	Cat#10-009-CV
Dulbecco’s Modified Eagle’s Medium (DMEM)	gibco	Cat#11995-065
Dulbecco’s Phosphate-Buffered Saline	Thermo Fisher Scientific	Cat#21-031-CV
DAPI (4′,6-Diamidino-2-Phenylindole, Dihydrochloride)	Thermo Fisher Scientific	Cat#D1306
Penicillin-Streptomycin (10,000 U/mL)	Thermo Fisher Scientific	Cat#15140122
L-Glutamine (200 mM)	gibco	Cat#25030081
Regular Fetal Bovine Serum	Corning	Cat#35010CV
16% Paraformaldehyde (formaldehyde) aqueous solution	Electron Microscopy Science	Cat#15710
Triton X-100	MilliporeSigma	Cat#T9284
Bovine Serum Albumin	MilliporeSigma	Cat#A9418
Normal Donkey Serum	Jackson ImmunoResearch	Cat#017-000-121
Normal Goat Serum	Cell Signaling Technology	Cat#5425S
Fc Receptor Blocker	INNOVEX biosciences	Cat#NB309-30
Trizol	Thermo Fisher Scientific	Cat#1596018

**Experimental models: Cell lines**		

Vero E6	ATCC	Cat#CRL-1586™
U118	ATCC	Cat#HTB-15™
HEK293 stably expressing TIM-1, AXL, DC-SIGN	UCLA^36^	N/A
Cortical brain organoids	UCLA^53^^,^^54^	N/A

**Experimental models: Organisms/strains**		

C57BL/6 female mice	Jackson Laboratories	Cat#000664

**Oligonucleotides**		

POWV Universal Primers	N/A	N/A
Forward: ACCATAACAAACATGAAAAGTCCAACT	N/A	N/A
Reverse: TGAGTCTGCTGGTCCGATGAC	N/A	N/A
GAPDH Primers	N/A	N/A
Forward: GTGGACCTGACCTGCCGTCT	N/A	N/A
Reverse: GGAGGAGTGGGTGTCGCTGT	N/A	N/A

**Software and algorithms**		

GraphPad Prism 10	GraphPad	N/A
ImageJ	ImageJ	N/A
MegAlign 15	DNASTAR Lasergene 15	N/A

### Experimental model and study participant details

#### Ethics statement

This study was performed in strict accordance with the recommendations of UCLA. All POWV and DTV live virus experiments were performed at the UCLA BSL-3 High Containment facility. The experiments were approved by the UCLA Institutional Biosafety Committee (#BUA-2017-263-001) and the UCLA Institutional Animal Care and Use Committee (#ARC-2017-068).^55^ This study does not involve human subjects and only uses human cells and cell lines. More specifically, iPSC lines generated by other investigators at UCLA were used for cortical organoid generation. We confirm that no one on this study team has the ability to link the data or specimens to human subject identifiable information. The de-identified human materials do not constitute human subjects research (45 CFR 46.102). SCRO-IRB application for use of human materials for our study is approved for the investigators.

#### Cell lines

Vero E6 cells (CRL-1586, ATCC) were cultured as monolayers in Minimum Essential Medium (MEM) supplemented with 10% fetal bovine serum (FBS), 2 mM L-glutamine and penicillin (100 units/ml), and streptomycin (100 units/ml), and were used for the propagation and titration of the three Powassan viral strains used in this study. U118 brain cells (HTB-15, ATCC) were cultured as monolayers in Dulbecco’s Modified Eagle’s Medium (DMEM) supplemented with 10% fetal bovine serum (FBS), 1X non-essential amino acids (NEAAs), penicillin (100 units/ml), and streptomycin (100 units/ml). 293T cells stably expressing TIM-1, AXL, and DC-SIGN were previously described.^36^ Human cortical organoids were generated as previously described.^53^^,^^54^ The 12-week-old cortical organoids were used in this study.

#### Viruses and virus titration

Lineage I POWV strain LB (LOT TC00580 WSV) isolated from a 5-year-old boy in Ontario, Canada in 1958^10^ and lineage II DTV WI_S_SPO_1997 [DTV (SPO)] strain isolated from the tick *Ixodes scapularis* in 1997 (collected by Gregory D. Ebel at Spooner, Wisconsin, USA), were obtained from Centers for Disease Control and Prevention (CDC). Additional lineage I Powassan Deer Tick virus (ONT_LT_M11665_1965; M11665), isolated from a tick *Ixodes cookei* in Ontario, Canada in 1965, was obtained from the Biodefense and Emerging Infections (BEI) Resources of the National Institute of Allergy and Infectious Diseases (NR-51182). Prior to distribution, these neurotropic viruses from human or tick biological samples were isolated by passaging in suckling mouse (SM) brain and/or Vero (V) cells.^35^ POWV LB strain from CDC had been passaged four times in suckling mouse brain and then twice in Vero cells (SM4V2). POWV M11665 isolate from BEI Resources was passaged twice in suckling mouse brain and three times in Vero cells (SM2V3). DTV SPO from CDC was passaged once in suckling mouse brain and two times in Vero cells (SM1V2). GenBank genome sequence accession numbers for POWV LB, POWV M11665, and DTV SPO are GenBank: NC_003687.1, GenBank: OP823404.1, and GenBank: OP823482.1, respectively. Note that the POWV LB strain is a highly passaged historic isolate, which may not reflect the circulating lineage I field isolate. Since we do not have the sequence information for these three strains directly from the clinical samples or tick extracts prior to passage in suckling mouse brain and cell culture, we cannot assess whether any specific cell-adaptive mutations are present. In our laboratory, POWV and DTV were passaged once in Vero E6 cells. Sequence-verified (Sanger sequencing) viral stocks were aliquoted and stored at −80°C. The viral titer was determined by measuring TCID_50_/0.1 mL (50% of Tissue Culture Infectious Dose) in Vero E6 cells at 37°C by a microtitration assay following the Spearman-Karber formula.^56^^,^^57^^,^^58^

### Method details

#### POWV and DTV infection

For POWV and DTV infection, U118 and 293T cells stably expressing DC-SIGN, AXL, and TIM-1 receptors were seeded in 48-well plates at approximately 50,000 cells per well. One day later, the cells were inoculated with all three POWV (LB), DTV (M11665), and DTV (SPO) viruses using an MOI of 1. Plates were incubated at 37 °C with 5% CO2 for 24 to 48 h post-infection (hpi). Uninfected cells were used as a negative control (Mock) in all the experiments. Mock control received the diluent (base media) used for diluting the virus.

#### Flow cytometry analysis of phosphatidylserine receptor expression in Vero E6 and U118 cells

Expression of TIM-1, AXL, TYRO3, MERTK and TIM-4 were analyzed in Vero E6 and U118 cells using flow cytometry. FACS buffer (PBS containing 2% FBS and 0.1% sodium azide) was used for staining and washing for flow cytometric analysis. Vero E6 or U118 cell (5 × 10^5^ cells) suspensions were stained with goat anti-human AXL, TYRO3, MERTK, TIM-1, and TIM-4 antibodies (bio-techne, Minneapolis, MN) or control goat IgG (bio-techne, Minneapolis, MN)) (2 μg/mL in 100 μL FACS buffer) for 30 min at room temperature. The cells were washed twice with 1 mL FACS buffer, followed by staining with PE-conjugated donkey anti-goat IgG antibody (Abcam, Cambridge, England) (2.5 μg/mL in 100 μL FACS buffer) for 30 min at room temperature. The cells were washed twice with 1 mL FACS buffer, followed by fixation with 2% paraformaldehyde (PFA) in FACS buffer. Flow cytometry analysis was performed using a LSRFortessa flow cytometer (BD Bioscience, San Jose, CA). 10,000 cell events were acquired per sample. FSC (Forward Scatter) and SSC (Side Scatter) plots with gating were generated using BD FACSDiva software. The expression level of each receptor protein is calculated by subtracting the mean fluorescent intensity (MFI) of the control antibody-stained cells from that of the cells stained with an antibody specific to a PtdSer receptor. A list of all antibodies used in this study is presented in key resources table.

#### Neutralization assay using a soluble form of TIM-1 (sTIM-1)

Vero E6 cells were plated 50,000 cells/well in 48-well plates and incubated overnight at 37°C. The next day, 100 plaque-forming units (PFU) (equivalent to 140 TCID_50_)^59^ from each viral strain were mixed with either 10 μg/mL of soluble TIM-1 receptor (sTIM1dMLDR801) or control (NCFcCQ R801) as negative treatment, then incubated for 30 min at room temperature. The mix was then used to inoculate naive Vero E6 cells for 1 h at 37°C, followed by wash and media replacement with complete media. 24 h later, the cells were collected with Trizol for RT-qPCR analysis. The sTIM1dMLDR801 and NCFcCQ R801 were produced as previously described.^43^

#### Mouse experiment

Five (*n* = 5) six-week-old C57BL/6 female mice were infected intraperitoneally with 1 × 10^4^ PFU (equivalent to 1.4 × 10^4^ TCID_50_)^60^ of DTV (SPO) while anesthetized with isoflurane. An additional 5 mice were inoculated with PBS and served as the negative control of the study. At 6 days post-infection (dpi), mouse brains were harvested in 4% PFA for immunohistochemistry.

#### Immunofluorescent staining

The cells were fixed for 30 min using 4% PFA, followed by 3 washes with PBS. Consequently, cell permeabilization was achieved by adding a blocking buffer containing 1% Triton X-100 and 5% FBS in PBS for 1 h at room temperature. All primary antibodies (key resources table) were appropriately diluted (1:100) in blocking buffer and incubated with cells overnight at 4°C. Cells were then washed 3 times with PBS, followed by the addition of the secondary antibodies (key resources table) diluted (1:250) in blocking buffer at room temperature for 1 h, protected from light. After the 1 h incubation with the secondary antibodies, cells were washed 3 times with PBS and incubated with DAPI (1:5,000) for 5 min at room temperature, protected from light. The cells were then examined under the fluorescent microscope for fluorescence.

Human cortical organoids were fixed in 4% PFA for 30 min, then washed 3 times with PBS, followed by the addition of 30% sucrose solution. The cortical organoids were then transferred and kept overnight at 4°C. The next day, after confirming sinking of the organoids to the bottom of the Eppendorf tube, they were embedded in optimal cutting temperature (OCT) compound and stored at −80°C until sectioning. A week later, the OCT blocks were sectioned to slides with 6 μm thickness using the Leica cryostat microtome and mounted on Superfrost precleaned microscope slides (VWR) or poly-*l*-lysine-coated German glass round coverslips (14 mm, no. 1) (Electron Microscopy Services). The slides were then washed with PBS one time for 10 min, and permeabilized using blocking buffer containing 1% Triton X-100 and 5% FBS in PBS for 1 h at room temperature. Primary antibodies diluted in blocking buffer were added to the slides and incubated overnight at 4°C. The next day, the organoids were washed 3 times with PBS, followed by addition of the secondary antibodies diluted in blocking buffer for 1 h at room temperature, protected from light. One hour later, the organoids were washed 3 times with PBS, and DAPI (1:5,000) was added for 5 min at room temperature, protected from light. All primary and secondary antibodies used in this study are presented in the key resources table.

#### Immunohistochemistry

Mouse brain tissues were fixed in 4% PFA for 1 h and then washed 3 times with PBS. Subsequently, the tissues were placed in 10% and 20% sucrose solution for 1 h, then transferred to 30% sucrose overnight at 4°C. The next day, tissues were embedded in OCT blocks and stored overnight at −80°C. Twenty-four (24) hours later, the tissues were cut (6–12 μm) using the Leica cryostat microtome and mounted on Superfrost precleaned microscope slides (VWR) or poly-*l*-lysine-coated German glass round coverslips (14 mm, no. 1) (Electron Microscopy Services). The sections were then washed with PBS one time for 10 min. Next, 500 μL of FC receptor blocker was added over each tissue sample for 60 min at room temperature. The tissues were then blocked for 1 h with blocking buffer (0.3% Triton X-100, 2% BSA, and 5% donkey and goat serum in 1× PBS) and permeabilized using primary antibody in blocking buffer overnight at 4°C. The next day, the tissues were washed 3 times with PBS, followed by addition of the secondary antibody diluted in blocking buffer (1:1,000). In the case of co-staining, secondary antibodies were added separately. Each secondary antibody was added for 1 h at room temperature. Sections were then rinsed with PBS, followed by the addition of DAPI (1:5,000) for 5 min at room temperature, protected from light. All primary and secondary antibodies used in this study are presented in the key resources table.

#### Confocal microscopy and image analysis

Confocal slide samples were imaged using the Leica SP8 MP-DIVE-FLIM Microscope at the Advanced Light Microscopy/Spectroscopy Laboratory and Leica Microsystems Center of Excellence at the California NanoSystems Institute at UCLA (RRID: SCR_022789), with funding support from NIH Shared Instrumentation Grant S10OD025017 and NSF Major Research Instrumentation grant CHE-0722519. Confocal three-dimensional images were collected in 1024 × 1024 format using a 63x oil immersion objective lens, fixed scan rate of 8000Hz, and averaged 12 times. Excitation laser lines and emission detection wavelengths were optimized for the fluorescent tags as follows: blue channel excitation of 405 nm with emission detection range of 420-470 nm, green channel excitation of 488 nm with emission detection range of 500 nm–530nm, and red channel excitation of 552 nm with emission detection range 590-650 nm.

#### RNA isolation, reverse transcription, and qPCR

RNA was extracted from the cells (100,000 cells) using a combination of the Trizol -chloroform RNA extraction protocol and the Aurum Total RNA Mini Kit (Bio-Rad, Hercules, CA). Usage of Trizol as lysis buffer ensures complete inactivation of the POWV since it is a BSL3 pathogen. The isolated RNA was then subjected to reverse transcription (1 μg RNA per reaction) using random primers and the Superscript III First strand synthesis system for RT-PCR (Invitrogen) in a 20 μL reaction volume. 1 μL of cDNA was subjected to qPCR using the SsoAdvanced Universal SYBR Green Supermix and the CFX384 touch real-time PCR detection system from Bio-Rad. Amplification was performed in a 10 μL final volume reaction using the 384-well plate format. The qPCR reaction was carried out as follows: initial denaturation at 95°C for 30 s (sec); 40 cycles in two steps: 95°C for 10 s, and 60°C for 1 min. At the end of the amplification cycles, melting temperature analysis was carried out from 65°C up to 95. Analysis of the data was performed by normalizing the POWV threshold sample (Ct) values to their respective glyceraldehyde 3-phosphate dehydrogenase (GAPDH) Ct for each sample. Utilizing the normalized POWV Ct values, the viral genome copy numbers were calculated. For this purpose, a standard curve was generated using 10-fold serial dilutions (10^1^-10^9^ copies) of a Powassan virus NS5 gene containing plasmid construct. We have also utilized the 2−^ΔCT^ method, and GAPDH threshold cycle (Ct) normalization for assessing genome replication. This approach was used to calculate fold changes in replication between different treatment conditions. The primers used in this study for the detection all of three viruses are universal POWV primers and are presented in the key resources table.

#### Western Blot analysis

To study the virus infection on a protein level, the cells were lysed on ice in 50 mM Tris pH 7.4, 1% NP-40, 0.25% sodium deoxycholate, 1 mM EDTA, 150 mM NaCl, 1 mM Na3VO4, 20 Mm or NaF, 1 mM PMSF, 2 mg mL^−1^ aprotinin, 2 mg mL^−1^ leupeptin and 0.7 mg mL^−1^ pepstatin or Laemmli Sample Buffer (Bio-Rad). The cell lysates were then resolved by SDS-PAGE using 10% gradient gels (Bio-Rad), followed by transferring to a 0.2 μm PVDF membrane (Bio-Rad). Membranes were then blocked (5% skim milk and 0.1% Tween 20) in 1x TBST (0.1% Tween 20) at room temperature (RT) for 1 h. The next step was the incubation with the respective monoclonal antibodies (1:1,000) overnight at 4°C. The next day the membranes were washed 3 times with TBST, followed by addition of HRP- conjugated antibody (1:5,000) for 1 h at room temperature. Subsequently enzymatic reaction on the membrane was initiated using the SuperSignal West Femto Maximum Sensitivity Substrate (Thermo Scientific). Finally, the membranes were exposed and visualized with the Bio-Rad ChemiDoc MP Imaging System.

### Quantification and statistical analysis

#### Genomic nucleotide sequence alignment for phylogenic tree and percent identity/divergence matrix construction

The following Powassan viral genomic sequences, GenBank: OP823482.1 (WI_S_SPO_1997), GenBank: OP823480.1 (WI_S_MB51273_2008), GenBank: OL695841.1 (PV320_2020), GenBank: NC_003687.1 (CAN_LB_1958), and GenBank: OP823404.1: (ONT_LT_M11665_1965) were retrieved from the NCBI Genebank database for sequence analysis. Note that the sequences GenBank: OP823480.1 (tick isolate, WI_S_MB51273_2008) and GenBank: OL695841.1 (human isolate, PV320_2020) were generated from viral isolates that did not undergo passaging in suckling mouse brain. The sequence information for the human isolate PV320 was obtained directly from BHK-21 cells infected with the patient’s cerebrospinal fluid. Multiple sequence alignment was performed using the MegAlign 15 module within DNASTAR Lasergene (Version 15), employing the ClustalW algorithm with default parameters. Based on the pairwise alignments, a phylogenetic tree was constructed using the Neighbor-Joining method (Saitou and Nei, 1987) with pairwise distance estimation. The matrix showing percent identity and divergence was also generated using the MegAlign 15 program.

### Statistical analysis

Statistical significance was analyzed using GraphPad Prism 10 by Student’s *t* test as reported in each figure legend. For two experimental groups, an unpaired Student’s *t* test was used. For all tests, *p* values were presented as ^∗^*p* < 0.05, ^∗∗^*p* < 0.01, ^∗∗∗^*p* < 0.001, and ^∗∗∗∗^*p* < 0.0001. The error bar stands for Standard Deviation (SD). A minimum of 3 biological replicates were used to calculate the standard deviation. Statistical details of each experiment can be found in the figure legends.
